# Assessment of the Factors Influencing the Patient's Comprehension of the Informed Consent to Interventional Pain Procedures

**DOI:** 10.1155/2023/7054089

**Published:** 2023-02-08

**Authors:** Mohammad Ghorbanhoseini, Kyle Kang, Allen Yang, Mohammadreza Abbasian, Eduard Vaynberg

**Affiliations:** ^1^Department of Anesthesiology, Boston Medical Center, Boston University School of Medicine, 750 Albany Street 2R, Boston 02118, MA, USA; ^2^Department of Anesthesiology, Boston Medical Center, Boston University School of Medicine, Boston, MA, USA; ^3^Postdoctoral Research Fellow, Musculoskeletal Translational Innovation Initiative, Beth Israel Deaconess Medical Center, Harvard Medical School, Boston, MA, USA; ^4^Chronic Pain Management, Boston University School of Medicine, Department of Anesthesiology and Pain Management, Boston Medical Center, Boston, MA, USA

## Abstract

**Background:**

Informed consent is the first step of every medical procedure and is considered a standard of care for patients undergoing medical interventions. Our study seeks to evaluate patients' understanding of the procedure they consented to and the factors affecting the degree of understanding.

**Methods:**

In this cross-sectional study, we used an anonymous postprocedural questionnaire to assess our patients' understanding of the procedure being performed and their level of satisfaction. It was conducted between June 2021 and January 2022 on every consenting patient who declined English interpreter services and was undergoing a first elective lumbar epidural steroid injection.

**Results:**

The mean age of 201 subjects was 57.3 (23–90) years, with a race distribution of Black (44.3%), White (31.8%), and other races (23.9%). 15.9% of our subjects worked in the medical field. Older age and patients identified as Black and other races had a positive correlation with the propensity to predict a poor understanding of consent. This study failed to demonstrate any difference in understanding of informed consent content between the different subgroups when stratified by assigned sex at birth, level of education, and profession. Patients' expectation from the treatment was classified as desperate (will take any help they can) in 78 patients (38.8%), feeling hopeful (expecting partial improvement in their symptoms) in 52 patients (25.9%), and being optimistic (will obtain full recovery from this injection) in 71 patients (35.3%). 192 patients (95.5%) were very satisfied with the consent process. Seven patients (3.5%) stated that they wanted more information, and 2 patients (1.0%) did not understand the explanation. 180 patients (89.6%) were satisfied with the overall experience, while 21 patients (10.4%) were not. The Wilks test (likelihood-ratio test) resulted in a *p* value of 0.023 and was deemed statistically significant for a relationship between understanding of consent and the satisfaction of the patient from the procedure.

**Conclusions:**

Although patients carry a variable expectation of procedures, most patients in our pain clinic have a high level of satisfaction despite having a poor understanding of the procedure provided via informed consent. Although our patients' level of objective comprehension is low, those with a better understanding of the procedure tend to have a more satisfactory experience.

## 1. Introduction

Informed consent is the first step of every medical procedure and is considered a standard of care for patients undergoing medical interventions. For informed consent to be valid, there are four criteria to be considered: adequate patient knowledge of the procedure, the possibility of patient-clinician communication, patient volunteerism, and capacity to consent [1–3].

It is recommended that the consent be written in a simple language for ease of comprehension by the maximum number of patients. At the same time, informed consent is a legal document and, therefore, can lead to a long form containing a large amount of specialized terminology and complicated information, making it difficult to interpret. In such a scenario, communication between the patient and the clinician becomes the cornerstone in optimizing the patient's knowledge of the prospective procedure and resolving vague aspects of treatment [1, 2, 4].

Before signing informed consent, most clinicians schedule a face-to-face interview with the patient to make sure that the procedure, alternatives, and complications are well understood. It has been proven that videos and other audiovisual aids significantly facilitate a patient's understanding and increase compliance with the follow-up treatment plan [3–7]. Several studies have shown significant improvement in the patient's information and satisfaction through interactions such as teach-back or test/feedback. It has been found that provider preparedness and utilization of verbal explanations, models, and reinforcement methods are much more effective than using information sheets or handouts while obtaining informed consent. Furthermore, it raises the level of awareness among patients, allowing them to feel more prepared and voice greater satisfaction (albeit not statistically significant) [3, 4, 8, 9].

Our study seeks to evaluate patients' understanding of the procedure they consented to and the factors affecting the degree of understanding. The assessment was conducted by a patient questionnaire filled out after the intervention. The primary outcomes are the patients' understanding of the procedure and factors affecting the outcomes and satisfaction.

## 2. Methods

To assess our patients' understanding of the procedure being performed, we implemented an anonymous postprocedural cross-sectional questionnaire in the recovery room that collected nonidentifying information and the level of understanding based on what we deemed basic knowledge regarding lumbar epidural steroid injections. This questionnaire study was conducted between June 2021 and January 2022 on all patients who declined English interpreter services and were undergoing their first elective lumbar epidural steroid injection for lumbar radiculopathy at a safety net tertiary care teaching hospital in Boston, MA. A consecutive patient sampling method was used.

The process would start at each patient's preprocedural evaluation by undergoing verbal explanations of a lumbar epidural steroid injection including preparations of the procedure, the local injection prior to epidural needle placement, descriptions of the epidural space (as a space next to the spine and not into the spine or muscle and bone around the spine), the sensation of the epidural needle (pressure sensation in contrast to a sharp pain), and complications from the injection and steroids (infection, bleeding, headache, and high blood sugar) via a standardized script presented by the same attending pain physician (EV, who was part of the study). Additionally, on the day of the procedure prior to proceeding, the consent process would be repeated in order to obtain standardized written informed consent. The time required for the consent process was around 3–5 minutes, including time for questions. No diagrams, videos, or leaflets were used or handed out. After the procedure, these patients, if willing, would be asked to fill out the anonymous questionnaire containing the following information: assigned sex at birth, age, ethnicity, level of education, and whether they are in the medical field or not. The questionnaire was self-administered by the patients in the recovery area.

To assess understanding of the procedure being performed, two multiple-choice questions regarding injection location and common complications were administered. (Attachment file) These questions were then graded on a scale of 0–5, obtaining one point for correctly identifying the injection location as a space next to the spinal cord and selecting each of the common complications we explained. Any wrong answers in the injection location question (as some patients selected more than one) were considered wrong. As for the question about complications, there were no points deducted for wrong answers. Satisfaction with the consent process and the procedure was also recorded, along with the expectation of the injection.

Approval by the institutional review board was granted. The requirement for individual consent for the study was waived by the IRB of the Boston University School of Medicine.

### 2.1. Outcome Measures and Statistical Analysis

The primary outcome was our patients' level of comprehension after our informed consent. We defined scores less than three as having a poor understanding of the procedure, while scores equal to or greater than three were considered to be having a good understanding. A score of 3 was selected as the cutoff point as it would be considered passing (60%). The patient's expectation of the procedure, level of discomfort, and satisfaction with the informed consent process and procedure were also recorded.

Statistical analysis was performed using IBM SPSS Statistic 26. Logistic regression was selected as our mode of analysis as we determined that comprehension could either be sufficient or lacking and decided that this binary result would be the most ideal for interventions in the future to be assessed upon. A future intervention that increases a patient's understanding by 20% would be vague and can be misinterpreted. Thus, our dependent variable was good and poor understanding. Age, assigned sex at birth, race, level of education, and whether they are in the medical profession were the variables to assess. The odds ratios and significance were recorded along with the 95% confidence interval. A *p* value of less than 0.05 was deemed statistically significant. The Hosmer–Lemeshow test was used to evaluate the goodness of fit for our model.

Our secondary outcome was our patients' satisfaction with the procedure, which was derived from the summation of the following patient's satisfaction with the consent process (scores of 0–2), level of discomfort experienced during the procedure (scores of 0–2), and pain relief level (scores of 0–3). A score of 4 out of 7 was selected as the cutoff as this was close enough to pass (57%), and the next value of 5/7 (71%) would be well over the passing score. This served as a surrogate marker for the quality of our informed consent and procedure. The data collected were also used to elicit a correlation between satisfaction and patient demographics. Analysis of the effect of the patient's subjective understanding on the patient's objective understanding and satisfaction was also conducted. Logistic regression and the Wilks test (likelihood-ratio test) were used in these instances.

## 3. Results

Our patients' baseline demographic data and distribution are presented in Table 1. The mean age of our 201 patients was 57.3 years, with a distribution ranging from 23 to 90 years. There were 111 females and 90 males.

Due to the small sample size of the race, education, and profession variables in some categories, we had to group and redefine some subcategories into one.

### 3.1. Level of Education

As there were only 12 patients with a master's (*n* = 10) or doctorate's (*n* = 2) degree, we decided to group them as a college + group (college or higher degree). Most of our patients had a high school degree (53.2%), followed by thosewho were in the college + category (34.8%) and a small percentage did not have any diplomas (11.9%).

### 3.2. Race

Due to the small percentage of those who self-identified as Asian (*n* = 9), American Indian (*n* = 2), multirace (*n* = 9), Latino (*n* = 24), and those who would rather not say (*n* = 3), we decided to cluster them into the group defined as other races (23.9%). The rest of the patients consisted of those who self-identified as Black (44.3%) and White (31.8%).

### 3.3. Profession

Due to the small number of medical workers among patients, we decided to consolidate the answers into yes/no regarding the profession. Our data showed that 15.9% of our patients work in the medical field, while most (84.1%) did not.

There were five answers, and all were weighted equally as described above. The distribution of the individual scores is listed in Table 2; however, as we consider “understanding” as an all-or-none phenomenon, we have decided to categorize our patient's understanding into a binary variable. A good understanding of the procedure was classified as answering more than half of the questions regarding the procedure correctly (score of 3), while those below three were denoted as having poor understanding. There was an overall poor understanding (71.1%) of the procedure in our sample population.

For our primary outcome, we performed logistic regression with the binary results of good and poor understanding while having a predictive outcome of poor understanding (a higher odds ratio would mean a higher likelihood of having poor understanding). With our determination of *p* < 0.05 as statistically significant, the analysis revealed race and age as independent predictors of poor understanding. Factors such as assigned sex at birth, level of education, and profession were not noted to be significant (Table 3). The goodness of fit was analyzed by the Hosmer–Lemeshow test and deemed significant with values greater than 0.05. Our value was 0.534, which reflects a good model fit.

### 3.4. Age

Data showed that age has a positive correlation with the propensity to predict poor understanding (*p* value ≤0.001) with a regression weight of +0.060 (or 6%) for every unit of age (every year older).

### 3.5. Race

We grouped the patients into three groups according to their answers: Black, White, and other races (Asians, Native Americans, Latinos, and mixed race). Compared to those who self-identify as White, patients who identify as Black and others have a 2.915 and 4.724 odds ratio, respectively, of predicting a poor understanding of the procedure. The *p* values were 0.010 and 0.002, respectively, and were deemed statistically significant.

At the end of our survey, we included questions pertaining to the patient's expectations of the procedure along with quality metrics such as patient satisfaction with the consent process and patient satisfaction with the experience (which included the patient's subjective level of discomfort during the procedure and satisfaction of the procedure).

### 3.6. Expectations

Overall, our patients were divided in terms of expectations, with 78 patients (38.8%) classified as desperate (will take any help they can), 52 patients (25.9%) feeling hopeful (expecting a partial improvement in their symptoms), and 71 patients (35.3%) being optimistic (will obtain full recovery from this injection).

### 3.7. Patient Satisfaction with the Consent Process

192 patients (95.5%) were very satisfied with the consent process. They stated that they received adequate information, and 7 patients (3.5%) stated that they wanted more information, while 2 patients (1.0%) stated that they did not understand the explanation.

### 3.8. Patient Overall Satisfaction with the Procedure

For overall satisfaction, we assigned a scoring system to all three questions and used the number of positive answers to determine a binary result of satisfaction (a total score of 0–4 was deemed to be not satisfied, while scores of 5–7 were deemed to be satisfied). 180 patients (89.6%) were noted to be satisfied with the overall experience, while 21 patients (10.4%) were not. The Wilks test (likelihood-ratio test) was then utilized for analysis and resulted in a *p* value of 0.023 and was deemed statistically significant (Table 4).

Overall, our survey showed that, although patients carry a variable expectation of procedures, most patients in our pain clinic have a high level of satisfaction despite having a poor understanding of the procedure provided via informed consent. Although our patients' overall level of objective comprehension is low, those with a better understanding of the procedure tend to have a more satisfactory experience.

### 3.9. Satisfaction with the Consent Process

We evaluated if our consent process was satisfactory to our patient population. 192 patients (95.5%) were very satisfied with the consent process and stated that they received adequate information regarding the procedure.

Unfortunately, despite the high satisfaction rate, further analysis revealed that only 58 out of the 192 patients satisfied with the consent process (30.2%) had a verifiable understanding of the procedure. Furthermore, of the 9 patients who wanted more information or did not understand our explanation, all were noted to have a poor understanding of and satisfaction with the procedure. The Wilks test was then conducted and showed that, for both patient satisfaction (*p* ≤ 0.001) and understanding (*p*=0.043), the patient's own subjective satisfaction and understanding of the consent process were noted to be predictive factors (Table 5). In this regard, we note the importance of repeated confirmation of a patient's subjective understanding. Not only does it affect their understanding objectively but also affects their overall satisfaction with the procedure.

### 3.10. Factors Influencing Comprehension of Informed Consent

To determine patient factors that correlate with patient understanding of the medical intervention, the following variables were accessed via regression analysis (Table 6).

## 4. Discussion

The process of informed consent involves the following elements: disclosure, comprehension, voluntary choice, and authorization [10]. The lack of comprehension breaks the chains of the process, thus altering the satisfaction with the intervention or potentially influencing the treatment results. Although the concept of consent was introduced over 100 years ago and the obligation to obtain consent by medical professionals has been present for over 50 years, repeated evaluation and analysis of our consent process, including our results, have shown that we are still falling short of adequately explaining interventions to our patients [11, 12].

### 4.1. Significant Factors: Age/Race

Patients' age and race showed a significant correlation with understanding of the informed consent content. Our analysis showed that both age (OR 1.060) and race (OR 2.915 comparing B/W and OR 4.724 comparing O/W) have statistical significance (*p* value of ≤0.001, 0.010, and 0.002, respectively) in predicting a poor understanding of the procedure. In previous research, it was shown that older age and ethnic background were linked to lower health literacy. Ashida et al. found a significant correlation between decreased health literacy and increased age [13]. Sarwat et al. found a strong association between race, health literacy, and access to care [14]. Our study corroborates these findings and further emphasizes the importance for pain physicians to be conscious of these variables, as they can affect the validity of informed consent, further negatively affecting patients' satisfaction and experience.

### 4.2. Nonsignificant Factors: Assigned Sex at Birth/Level of Education/Profession

This study failed to demonstrate any difference in understanding of informed consent content between the different subgroups when stratified by assigned sex at birth, level of education, and profession variables. Although there were tendencies in comparing different groups with each other for predicting a poor understanding of the consent, such as male gender compared to female gender (odds ratio 1.29), those without diploma groups compared to those with college or higher degrees(odds ratio 3.16), those with high school diplomas compared to those with college or higher degrees (odds ratio 1.39), and being in the field of medicine compared to those not in the field of medicine (odds ratio 1.36), none of them were statistically significant (*p* value of 0.492, 0.072, 0.601, and 0.524 respectively). These results contrast previous research findings that men, with lower socioeconomic status, and lower educational attainment are associated with lower levels of health literacy [15–19]. In this aspect, our study could not replicate these results regarding the 7 levels of understanding of informed consent. We attribute these results to possible inadequate study power given a limited number of enrolled patients with advanced educational degrees.

### 4.3. The Possible Presence of Affinity Bias

Irrespective of the result of patient understanding, we also ran logistic regression using patient factors to predict the incidence of patient satisfaction (Table 6). The Hosmer–Lemeshow test in this scenario also predicts a good fit for our logistic regression model.

Interestingly, the variables that were shown to have a predictive value were assigned sex at birth, B/W race, and medical profession. Females, who self-identify as Black, compared to those who self-identify as White, and who did not work in the medical field, were more likely to have poor satisfaction with the procedure. One of the possible explanations is the presence of the affinity bias as a pain physician obtaining informed consent and performing the procedure in our study is a male who self-identifies as White.

### 4.4. Improving Informed Consent

Our study demonstrates the need for improvement in the process to ensure better comprehension of informed consent among interventional pain patients. Nowadays, due to the increasing complexity of care and legal implications, standard informed consent documents are often written above the population's average literacy level [20]. As patients with lower health literacy levels are associated with lower comprehension [14], studies utilizing a modified or simplified consent form were thought to improve comprehension. However, this was not shown to be accurate, and we have only reaffirmed the fact that a patient's health literacy level affects comprehension [21, 22].

Where do we go from here? With a diverse patient population and varying health literacy levels, particularly at safety net hospitals, informed consent must be viewed as a continuous dynamic process rather than an isolated event to aid in knowledge assimilation [2]. This is something we have tried to tackle, albeit unsuccessfully, as we now repeat our explanation process multiple times at multiple visits, allowing abundant opportunities and time to ask questions. Furthermore, as utilizing modified and simplified consent forms has been unsuccessful, we must seek out other modalities to enhance comprehension of informed consent. With technological advances, multimedia tools have been shown to improve components of informed consent [23, 24]. Tipotsch-Maca et al. showed that, by adding a multimedia presentation in the form of a computer-animated video, there was an increase in information retention even in patients of increasing age [24]. With age being one of the significant factors impacting the level of comprehension, this may be the next step in providing high-quality informed consent. With the best of intentions in mind, we must also be aware of cultural variations and expectations of our patients and possibly open the floor up and request aid from our nursing colleagues to be part of the consent process, as some may feel that they are easier to approach [25].

While all these changes are being thought of, we still have to be conscious of all the other factors that predict poor understanding such as race (as in our study) and possibly health literacy. Specifically, those who self-identify as Black not only appear to have an overall lower rate of understanding but also seem to have a poor satisfaction rate when compared with patients who self-identify as White. The reason behind this is likely multifactorial which stems from a long-standing history of distrust in the healthcare system and the potential for affinity bias, and we must be cognizant of such factors if we are to improve healthcare management and outcomes within this population [26]. The use of multimedia in the process of informed consent could alleviate affinity bias and needs further study.

### 4.5. Limitations

There are a number of potential limitations in our study. The relatively small sample size may not have adequate statistical power to expose slight differences resulting in type II errors. Our small sample size also led us to consolidate different subgroups together such as other races in the race variable, C+ in the level of the education variable, and the yes group in the profession variable. This process most likely caused this study to lose its ability to generalize to these respective populations. It is possible that the distribution within these subgroups in our study does not match that of the general population.

Furthermore, due to our selection of only English-proficient patients and the voluntary nature of filling out questionnaires, only willing patients who were comfortable speaking and reading English filled out the survey after each procedure. There might be intrinsic bias in our sample as we suspect those more willing may be those who were more satisfied, and affinity bias might have played a role in patient satisfaction.

## 5. Conclusion

Our study indicates there is a poor level of understanding of the interventional pain procedures of the informed consent process among the majority of patients at the tertiary care safety net hospital, indicating a need to enhance the consent process. The initial goal of informed consent to improve patients' comprehension and satisfaction with the interventional pain procedure seems to be poorly met at present via the accepted methodology. As multiple factors seem to affect our patients' comprehension of the ever-growing complexity of medical care, physicians must remain mindful of these factors at all times. Further study of novel ways of obtaining informed consent besides verbal preprocedure discussion to eliminate bias and improve comprehension is warranted.

## Figures and Tables

**Table 1:** Demographic distribution of the patients (*n* = 201).

Demographic data	Category and distribution [*n*] %		
Assigned sex at birth	Female (111) 55.2%	Male (90) 44.8%	
Age range	23 years old–90 years old		

Degree	ND (24) 11.9%	HS (107) 53.2%	College+ (70) 34.8%
Race	Black (89) 44.3%	White (64) 31.8%	Others (48) 23.9%
Medical profession	No (169) 84.1%	Yes (32) 15.9%	

**Table 2:** Patients' objective understanding based on the postprocedure score.

Score	*n* (%)	Objective understanding
0	42 (20.9%)	Poor understanding 143 (71.1%)
1	45 (22.4%)	
2	56 (27.9%)	

3	29 (14.4%)	Good understanding 58 (28.9%)
4	24 (11.9%)	
5	5 (2.5%)	

**Table 3:** Relationship between patients' demographics and their objective understanding of the procedure, with the propensity to predict poor understanding.

Variables		OR	95% CI	*p* value^a^
Assigned sex at birth (female : male)		0.776	0.375–1.602	0.492

Age [23–90]		1.060	1.028–1.093	**≤0.001**

Degree	(C+ : ND)	0.316	0.090–1.110	0.072
	(HS : ND)	0.721	0.211–2.461	0.601

Race	(B : W)	2.915	1.294–6.564	**0.010**
	(O : W)	4.724	1.750–12.749	**0.002**

Medical profession (no : yes)		0.726	0.271–1.945	0.524

OR, odds ratio; CI, confidence interval; C+, college and higher degrees; ND, no diploma; HS, high school; B, Black; W, White; O, other races including Latino, American Indian, Asian, and multirace. ^a^Binary logistic regression. ^b^Hosmer–Lemeshow test: chi-square 7.024 with significance 0.534. Bold values are statistically significant.

**Table 4:** Objective understanding and satisfaction.

Understanding	Poor satisfaction (0–4)	Satisfied (5–7)	Total
Poor	19 (12.0%)	124 (88.0%)	143
Good	2 (3.6%)	56 (96.4%)	58
*p* value: 0.023^a^			

^a^Wilks test (likelihood-ratio test).

**Table 5:** Effect of patient's subjective understanding on objective understanding and satisfaction.

Answers	Objective understanding^a^			Satisfaction^b^		
	Poor (%)	Good	Total	Poor (%)	Good	Total
Did not understand	2 (100)	0	2	2 (100)	0	2
Would have liked more information	7 (100)	0	7	7 (100)	0	7
Enough information	134 (69.8)	58 (30.2)	192	12 (5.3)	180 (94.7%)	192
Total	143 (71.1)	58 (28.9%)	201	21 (10.4)	180 (89.6%)	201
Significance	*p* value: 0.043^c^			*p* value: ≤0.001^c^		

^a^Scores of 0–2 classified as poor understanding and 3–5 as good understanding. ^b^Scores of 0–4 classified as poor satisfaction and 5–7 as satisfied. ^c^Wilks test (likelihood-ratio test).

**Table 6:** Relationship between patient's demographics and their satisfaction of the procedure, with the propensity to predict poor satisfaction with the procedure.

Variables		OR	95% CI	*p* value^a^
Assigned sex at birth (female : male)		0.334	0.120–0.928	**0.035**

Age [23–90]		0.992	0.952–1.034	0.711

Degree	(C+ : ND)	1.792	0.321–9.991	0.506
	(HS : ND)	1.208	0.223–6.549	0.826

Race	(B : W)	3.917	1.123–13.656	**0.032**
	(O : W)	1.528	0.326–7.165	0.591

Medical profession (no : yes)		0.194	0.066–0.574	**0.003**

OR, odds ratio; CI, confidence interval; C+, college and higher degrees; ND, no diploma; HS, high school; B, Black; W, White; O, other races including Latino, American Indian, Asian, and multirace. ^a^Binary logistic regression. ^b^Hosmer–Lemeshow test: chi-square 6.688 with significance 0.571. Bold values are statistically significant.

## Data Availability

Data are available on request from the corresponding author.

## Supplementary Materials

Supplementary Materials The questionnaire that was used for this study.
