# Supplementary material for: Assessment of the Factors Influencing the Patient's Comprehension of the Informed Consent to Interventional Pain Procedures
Source: Pain Res Manag. 2023 Feb 8;2023:7054089. doi: 10.1155/2023/7054089 (PMC9931461; doi:10.1155/2023/7054089)
Supplement: Supplementary Materials — The questionnaire that was used for this study. [file 7054089.f1.pdf]

RESEARCH INFORMATION SHEET

You are being asked to voluntarily participate in a research study. We are doing this study as the pain clinic of the Department of Anesthesiology at Boston Medical Center is interested in learning how well patients comprehend the procedure performed, possible complications, potential effectiveness of the treatment, and their overall satisfaction. If you agree, we will ask you to fill up the questionnaire below, it will take less than 5 minutes.

We will not record your name or any identifying information. You will not be signing this form. A completed form will be placed in the sealed envelope, collected by a third party, and opened at the conclusion of the study.

If you have any questions, please contact Dr. Eduard Vaynberg, email: eduard.vaynberg@bmc.org, and phone number 617-638-6950

1- Assigned sex at birth Male □ Female □

2- Age ……

3- Education:

□ Some school, no diploma
□ High School graduate, diploma or the equivalent
□ College/Bachelor's degree
□ Master's degree
□ Doctorate's degree

4- What is your ethnicity?

□ Latino
□ White
□ Black or African-American
□ American Indian or Alaskan Native
□ Asian
□ Native Hawaiian or other Pacific Islander
□ From multiple races
□ Would rather not say

5- Do you work in the medical field?

□ No
□ Administrator/non-clinical personnel
□ Medical Assistant
□ Nurse/physician extender
□ Physical/Occupational Therapist
□ Physician
□ Other: ________________________

6- What best describes the treatment you got?

□ An injection into the bones of your spine
□ An injection into your spinal cord
□ An injection into space near your spinal cord
□ An injection into the muscles in your back
□ I don't exactly know

7- What are the most common complications after this procedure? (You can choose more than one answer)

□ Bleeding
□ Infection
□ Paralysis
□ Muscle twitching
□ Headache
□ High blood sugar
□ Rash

8- How satisfied are you with your injections?

□ Very satisfied
□ I got partial relief
□ They did not work for me
□ This is my first injection

9- Was the level of discomfort during the procedure as you expected?

□ Yes, as described
□ More painful than expected
□ Less painful than expected

10- How satisfied are you with the explanation given to you during the consent?

□ Very satisfied, I got enough information
□ I could have been given more information
□ I didn't understand what they told me

11- What is your expectation from getting these injections?

□ I expect full recovery from the pain
□ I expect partial recovery from pain
□ I will take any help I can get from these injections
□ I don't think these injections will help, let's see what happens
